# Supplementary material for: Conformational isomerism breaks the electrolyte solubility limit and stabilizes 4.9 V Ni-rich layered cathodes
Source: Nat Commun. 2024 Oct 22;15:9108. doi: 10.1038/s41467-024-53570-1 (PMC11496818; doi:10.1038/s41467-024-53570-1)
Supplement: Supplementary file 1 — Supplementary Information [file 41467_2024_53570_MOESM1_ESM.pdf]

Supplementary Information for

Conformational isomerism breaks the electrolyte solubility limit and stabilizes 4.9 V Ni-rich layered cathodes

Ziyang Lu[1,2,3], Huijun Yang[2,3], Jianming Sun[1,2], Okagaki Jun[2], Yoongkee-Choe[2] and Eunjoo Yoo[1,2]*

[1]Graduate School of System and Information Engineering, University of Tsukuba, 1-1-1, Tennoudai, Tsukuba 305-8573, Japan.

[2]Energy Technology Research Institute, National Institute of Advanced Industrial Science and Technology (AIST), 1-1-1, Umezono, Tsukuba 305-8568, Japan.

[3]These authors contributed equally.

Correspondence to: yu.eunjoo@aist.go.jp (E. Yoo)

Inventory of Supporting Information

- Supplementary Figures S1 to S28
- Supplementary Tables S1

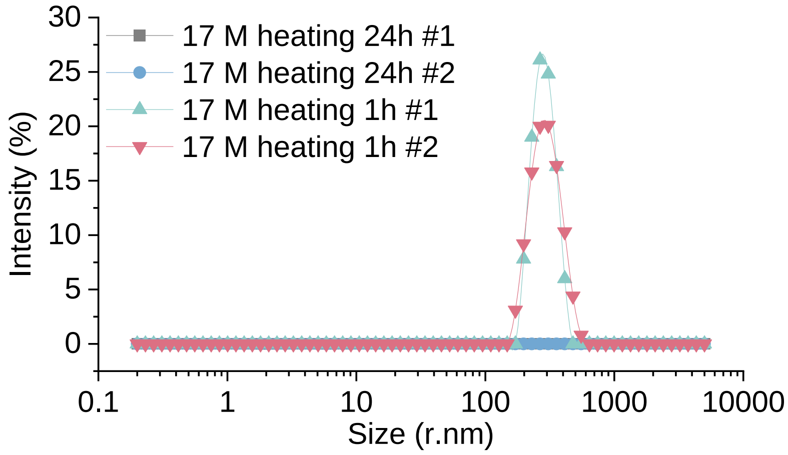

Supplementary Figure 1 | Stability of electrolytes after different heating time. Dynamic light scattering (DLS) analysis for the 17 M LiFSI-DMC electrolytes after heating 24 h and 1 h.

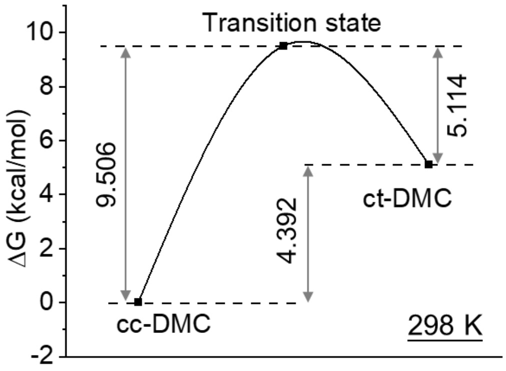

Supplementary Figure 2 | Calculated free energy diagram for cc-DMC, ct-DMC and corresponding transition state at 298 K. This figure clearly shows the energy barrier that needs to be crossed to transform between the two configurational isomers.

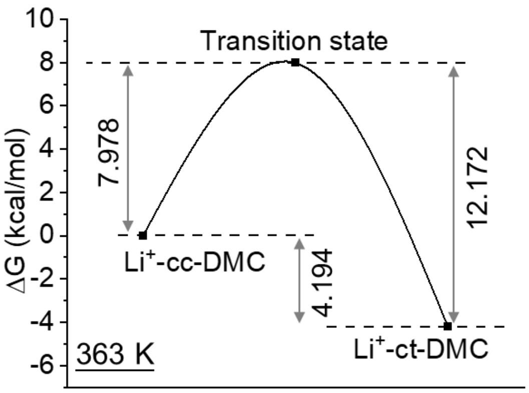

Supplementary Figure 3 | Calculated free energy diagram for Li^+-cc-DMC, Li^+-ct-DMC and corresponding transition state at 363 K. This figure clearly shows the energy barrier that needs to be crossed to transform between the two configurational isomers in the presence of Li^+.

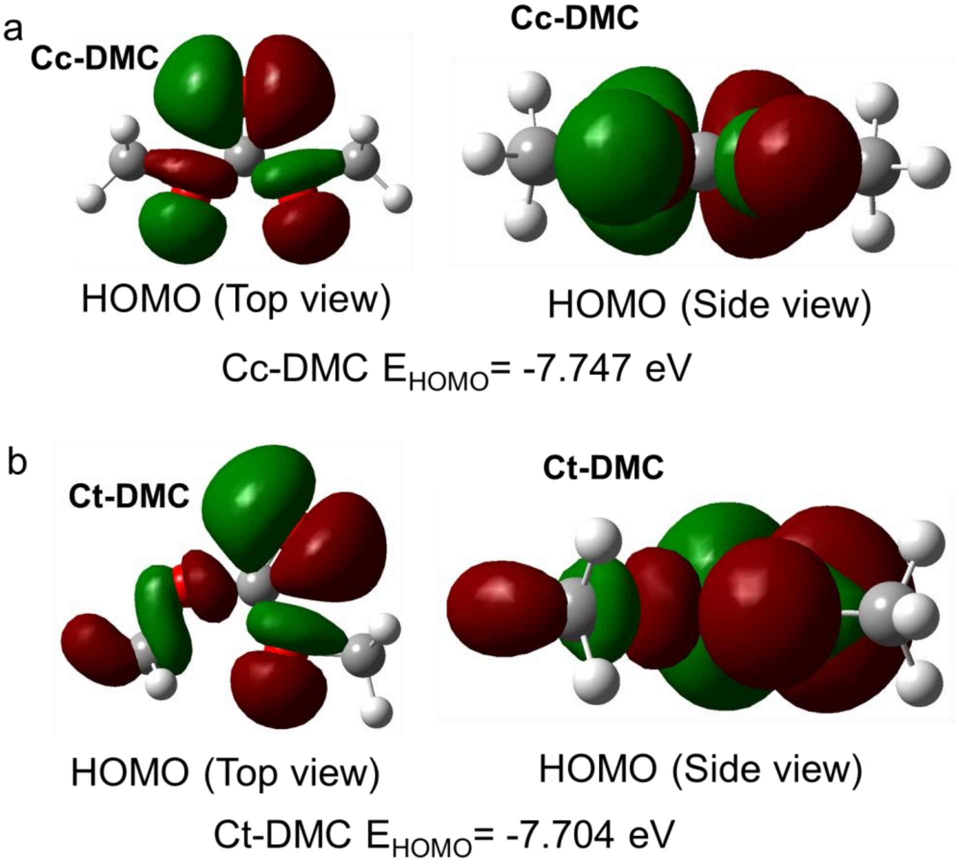

Supplementary Figure 4 | The structures of HOMO for cc-DMC and ct-DMC. a-b, Top view and side view HOMO structures of the cc-DMC (a) and ct-DMC (b).

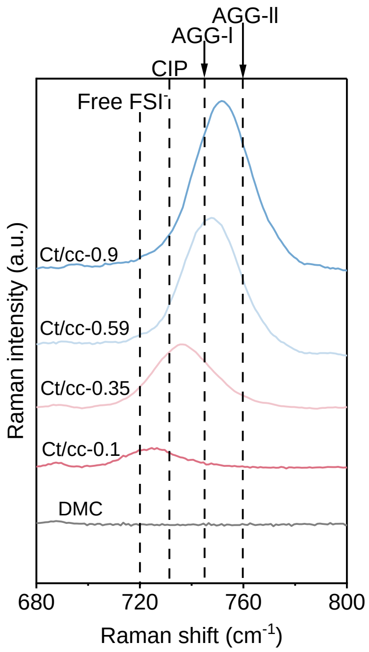

Supplementary Figure 5 | The Raman spectra of various electrolytes. The S-N stretching of the FSI^- for electrolytes with different concentrations.

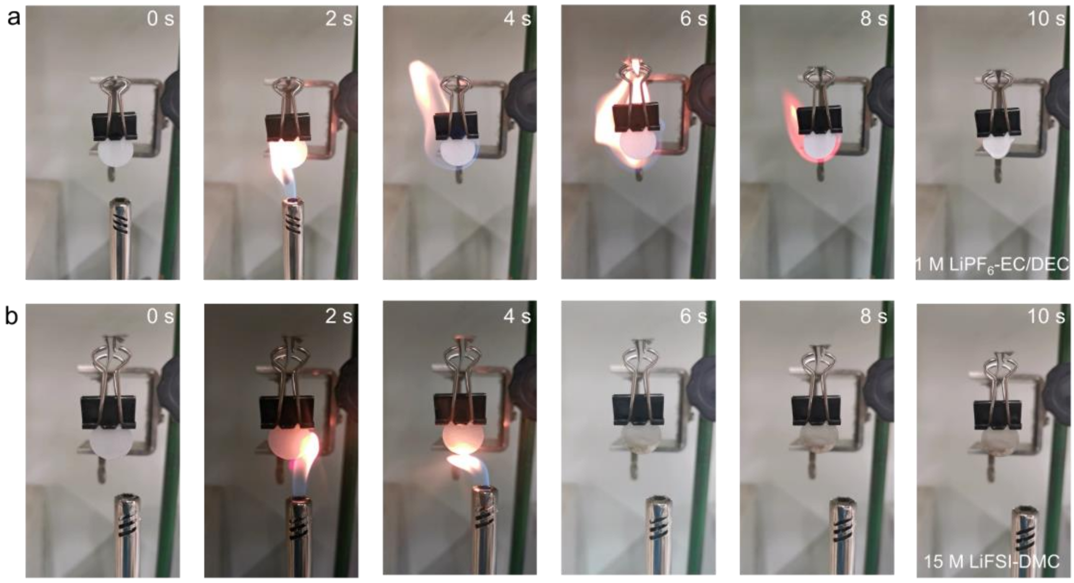

Supplementary Figure 6 | Electrolyte flammability test. a-b, Flame tests of commercial 1 M $LiPF_6$-EC/DEC electrolytes (a) and 15 M LiFSI-DMC electrolytes (b).

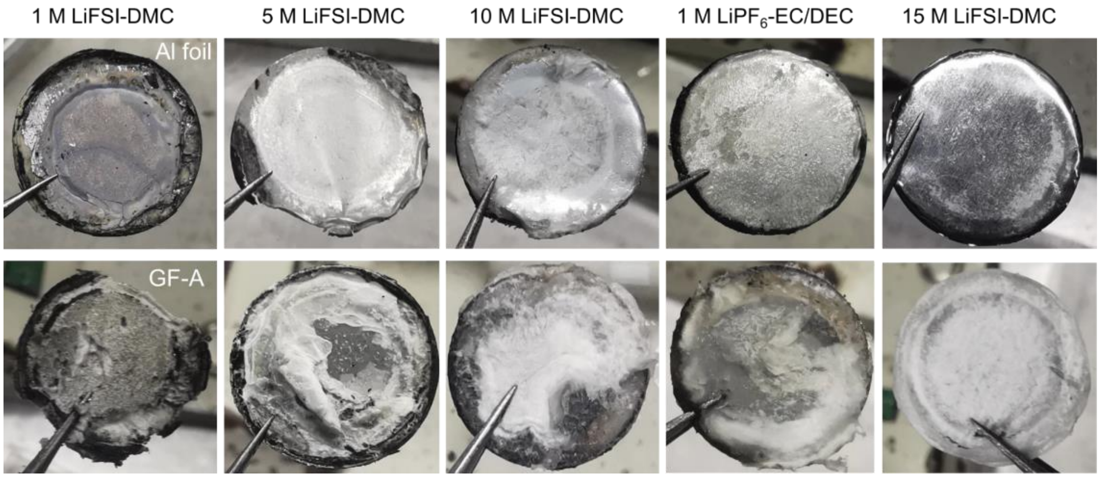

Supplementary Figure 7 | Demonstration of corrosion degree of Al current collectors and GF-A separators using different electrolytes. Photographs of Al foil and glass fiber separator cycled in Li||Al cells with different electrolytes after LSV test and aging for 1 month.

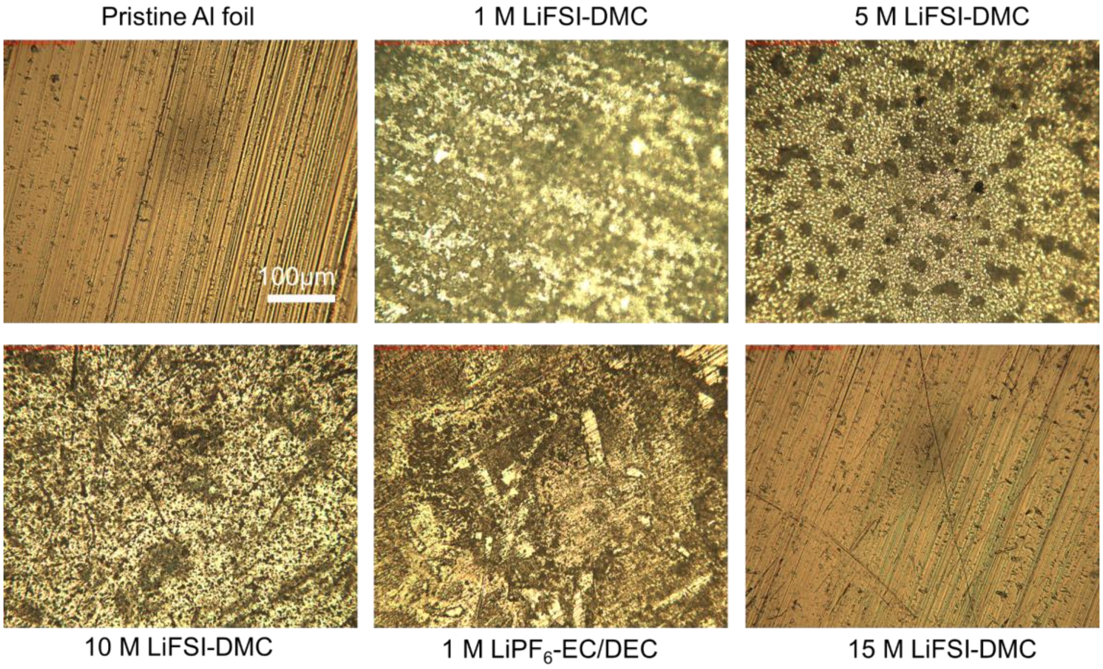

Supplementary Figure 8 | The stability of Al current collectors. Optical microscope images of Al foil cycled in Li||Al cells with different electrolytes after LSV test and aging for 1 month.

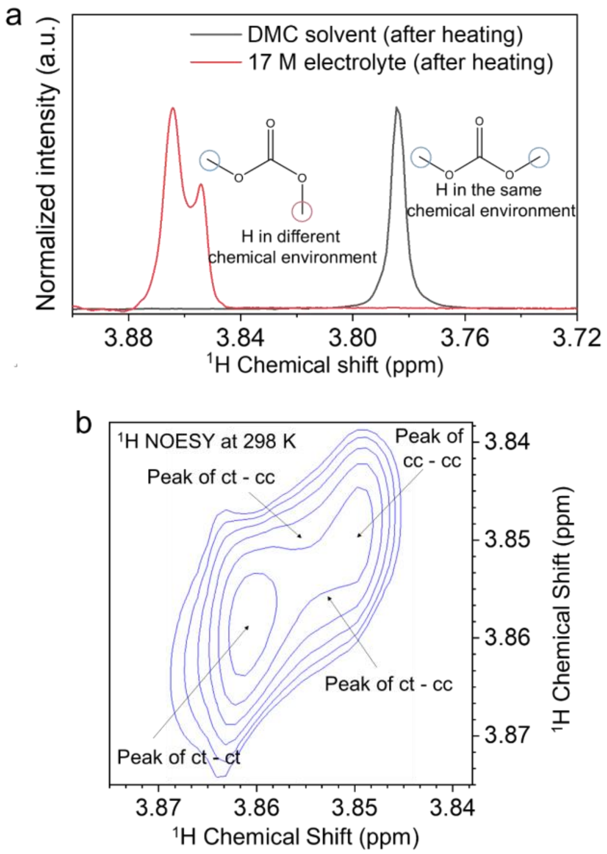

Supplementary Figure 9 | NMR spectra of ^{1}H and NOESY NMR spectra. a, ^{1}H spectra of 17 M electrolytes and DMC solvent at temperature after returning to 298 K. **b**, The fragment of ^{1}H–^{1}H NOESY NMR spectrum of 17 M electrolytes after returning to 298 K.

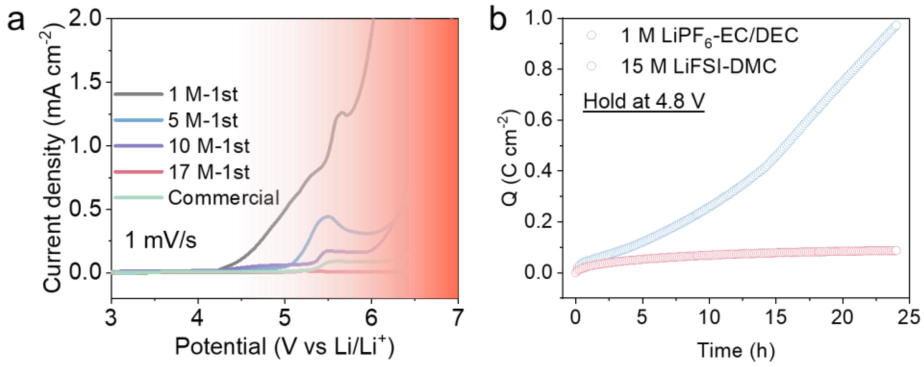

Supplementary Figure 10 | The high voltage stability of various electrolytes. a, LSV of Li||Al cells in various concentrations of LiFSI/DMC electrolytes. **b**, Potentiostatic test at 4.8 V for ct/cc-0.82 (15 M LiFSI-DMC) electrolytes and 1 M $LiPF_6$-EC/DEC electrolytes. As a standard two-electrode battery configuration, Li foil was used as the reference and counter electrode, and the Al foil used as the working electrode.

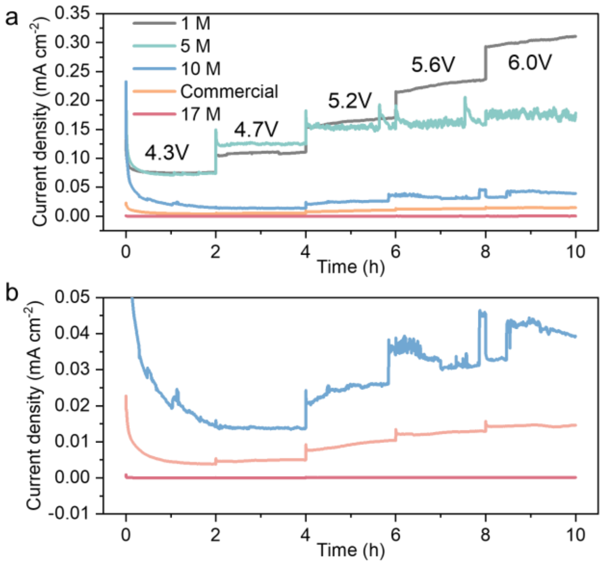

Supplementary Figure 11 | The high voltage stability of various electrolytes at different potentials. a, Potentiostatic Intermittent Titration Technique floating test of different electrolytes in Li||Al cells. **b**, The enlarged region for 10 M, 17 M LiFSI-DMC and commercial electrolytes. As a standard two-electrode battery configuration, Li foil was used as the reference and counter electrode, and the Al foil used as the working electrode.

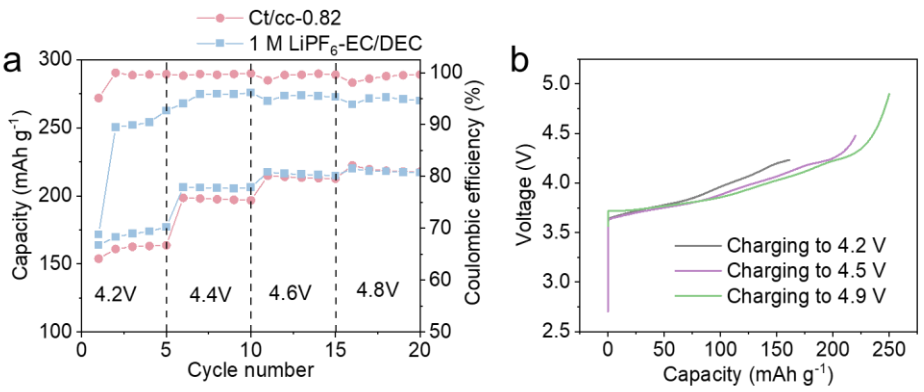

Supplementary Figure 12 | Capacity comparison under different charging voltages for NCM811 cathode. a, Capacity comparison at different charging cut-off voltages using 1 M $LiPF_6$-EC/DEC electrolytes and ct/cc-0.82 electrolytes. The mass loading for the NCM811 is about 1.3 mg cm^{-2}. **b**, Charging curves under different charging cut-off voltages for the battery with ct/cc-0.82 electrolytes.

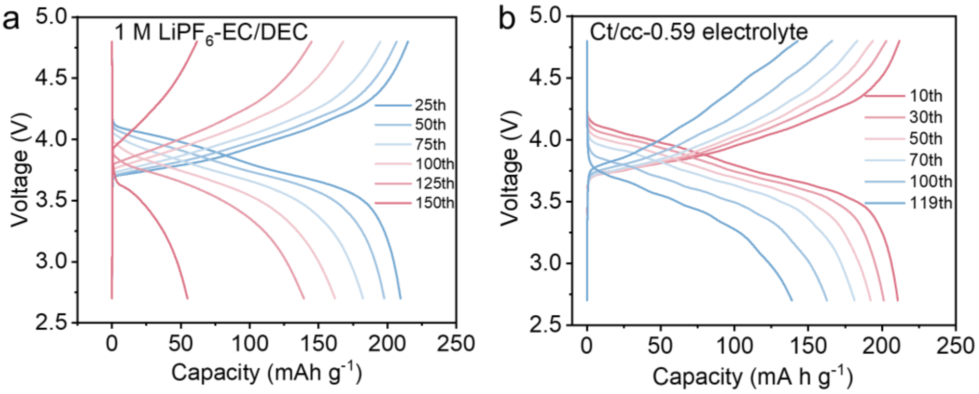

Supplementary Figure 13 | Selected charge-discharge curves of Li||NCM811 battery with cut-off voltage of 4.8 V. a, In 1 M $LiPF_6$-EC/DEC electrolytes. **b**, In ct/cc-0.59 electrolytes. The mass loading for the NCM811 is about 1.3 mg cm^{-2}, and the current density applied here is 0.5C (100 mA g^{-1}).

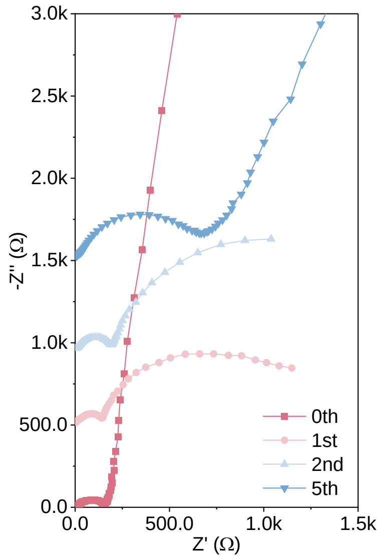

Supplementary Figure 14 | EIS results of Li||NCM811 cells within 2.7~4.9 V using 1 M $LiPF_6$-EC/DEC electrolytes. The EIS tests are conducted after different cycles. The interfacial impedance increases significantly with cycling.

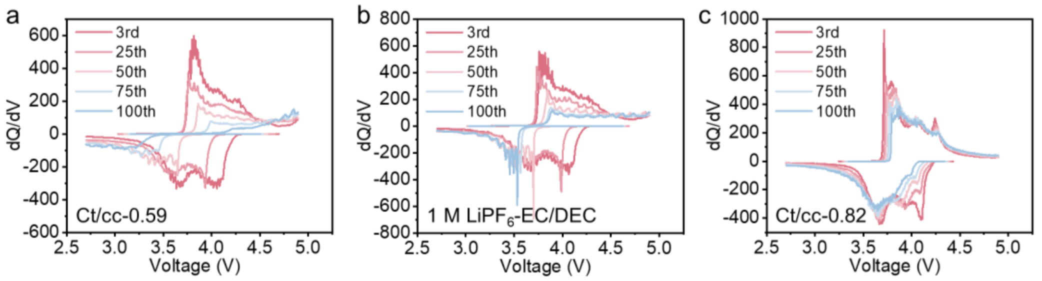

Supplementary Figure 15 | Selected dQ/dV curves of 4.9 V-class Li||NCM811 batteries with different electrolytes. **a**, Ct/cc-0.59 electrolytes. **b**, 1 M $LiPF_6$-EC/DEC electrolytes. **c**, Ct/cc-0,82 electrolytes. The mass loading for the NCM811 is about 1.3 mg cm^{-2}, and the current density applied here is 0.5C (100 mA g^{-1}).

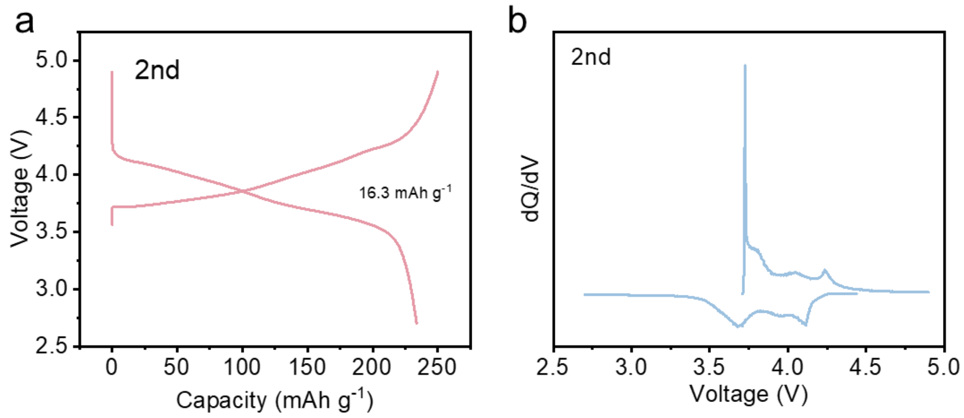

Supplementary Figure 16 | Electrochemical characteristics of Li||NCM811 cells using ct/cc-0.82 electrolyte at 2nd cycle. a, Discharge/charge curves. **b**, The corresponding dQ/dV curves. The mass loading for the NCM811 is about 1.3 mg cm^{-2}, and the current density applied here is 0.5C (100 mA g^{-1}).

a

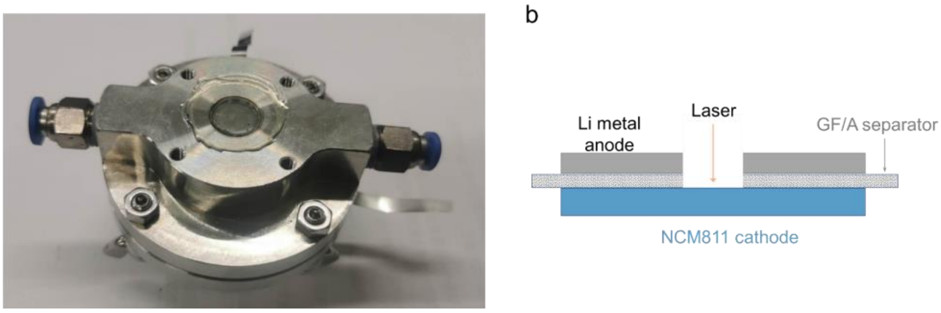

Supplementary Figure 17 | The battery configuration for operando Raman test. a, The photo of customized equipment for operando Raman test. b, Schematic diagram of the cell configuration for operando Raman test.

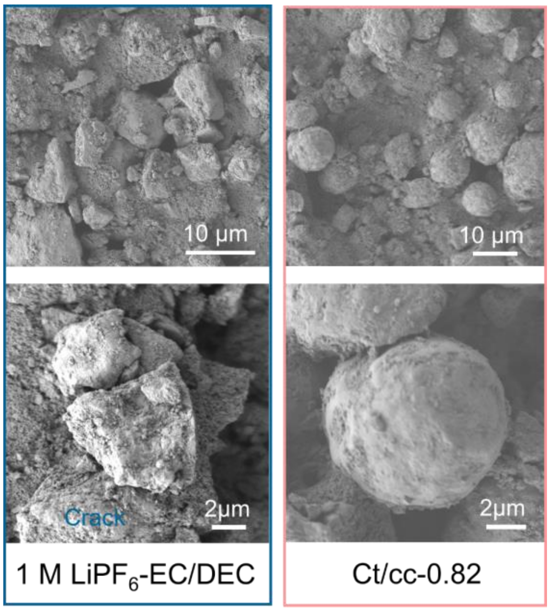

Supplementary Figure 18 | SEM images of cycled NCM811 cathode in 1 M $LiPF_6$-EC/DEC electrolytes and ct/cc-0.82 electrolytes. The NCM811 cathode was harvested after cycling for 68 cycles at current density of 0.5C (100 mA g^{-1}), and the cathode is in a fully discharged state. The charging cut-off voltage is 4.8 V.

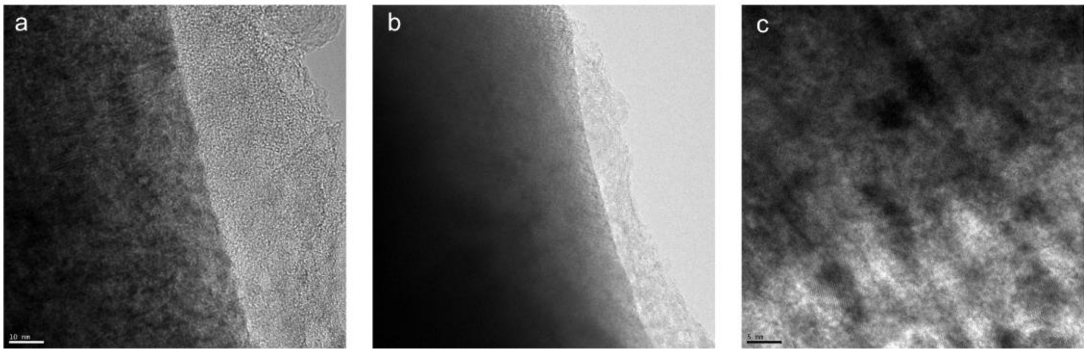

Supplementary Figure 19 | TEM images of NCM811 cathode cycled in 1 M $LiPF_6$-EC/DEC electrolytes. a-c, The NCM811 cathode was harvested after cycling for 68 cycles at current density of 0.5C (100 mA g^{-1}), and the cathode is in a fully discharged state. The charging cut-off voltage is 4.8 V.

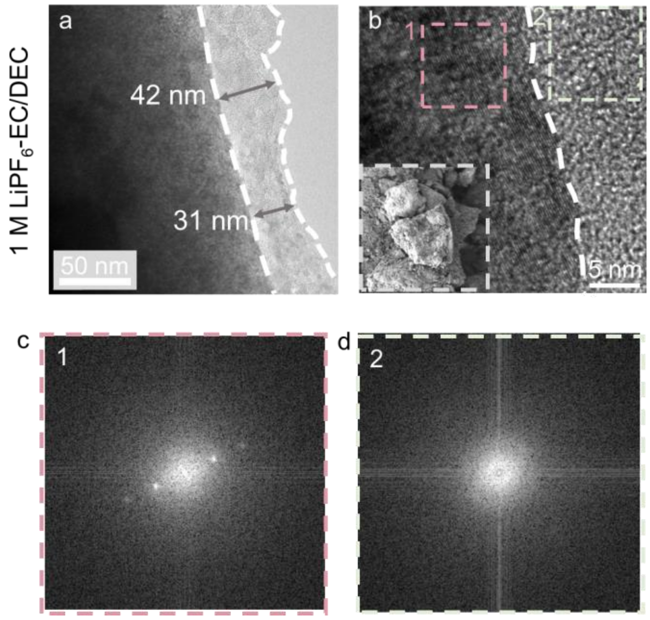

Supplementary Figure 20 | Characterization of CEI formed in 1 M $LiPF_6$-EC/DEC electrolytes. a-b, The TEM images of NCM811 cathodes harvested from battery using 1 M $LiPF_6$-EC/DEC electrolytes. **c-d,** FFT images of the region outlined in Supplementary Figure 19b. The NCM811 cathode was harvested after cycling for 68 cycles at current density of 0.5C (100 mA g^{-1}), and the cathode is in a fully discharged state. The charging cut-off voltage is 4.8 V.

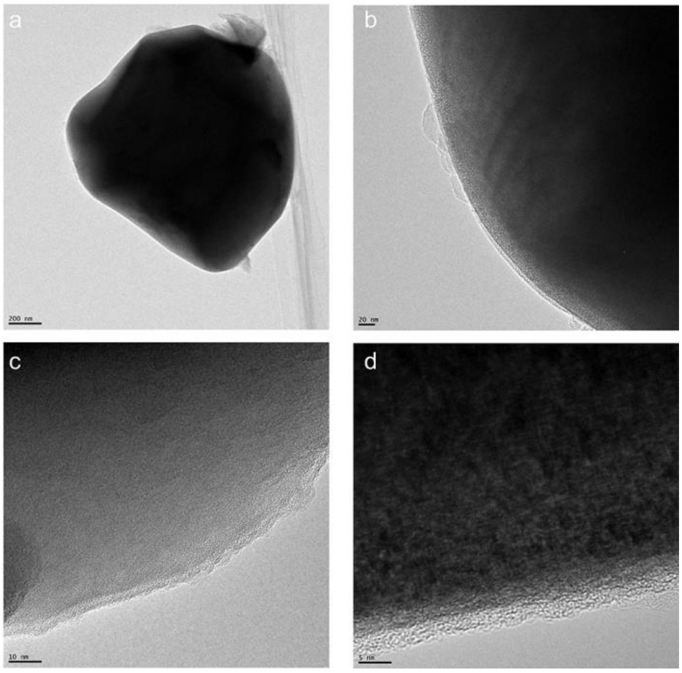

Supplementary Figure 21 | Characterization of CEI formed in ct/cc-0.82 electrolytes. a-d, TEM images of NCM811 cathode cycled in ct/cc-0.82 electrolytes. The NCM811 cathode was harvested after cycling for 100 cycles at current density of 0.5C (100 mA g^{-1}), and the cathode is in a fully discharged state. The charging cut-off voltage is 4.8 V.

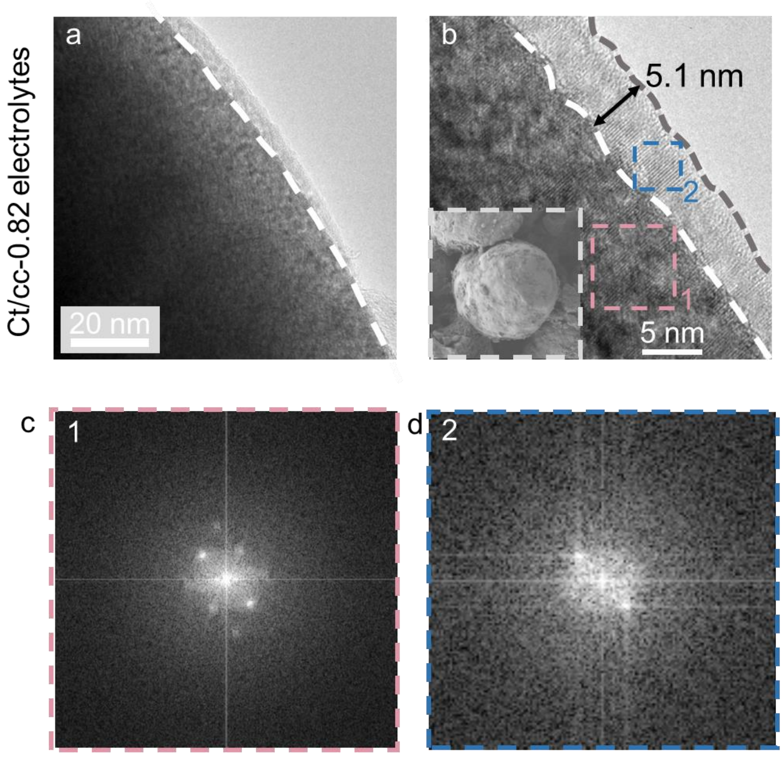

Supplementary Figure 22 | Characterization of CEI formed in ct/cc-0.82 electrolytes. a-b, The TEM images of NCM811 cathodes harvested from batteries using ct/cc-0.82 electrolytes. **c-d,** FFT images of the region outlined in Supplementary Figure 21b. The NCM811 cathode was harvested after cycling for 100 cycles at current density of 0.5C (100 mA g^{-1}), and the cathode is in a fully discharged state. The charging cut-off voltage is 4.8 V.

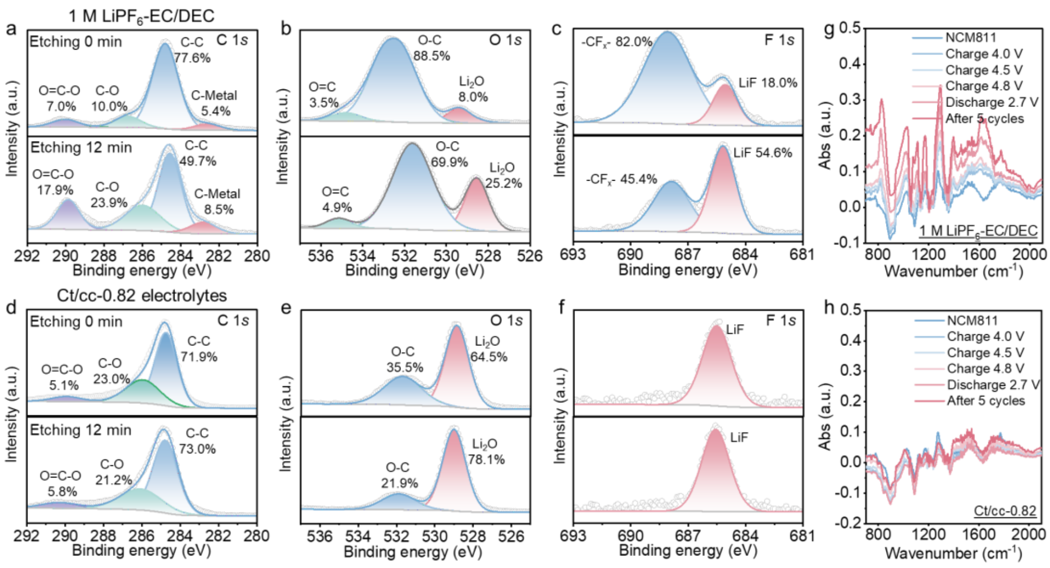

Supplementary Figure 23 | CEI analysis of NCM811 cycled in 1 M LiPF6-EC/DEC and ct/cc-0.82 electrolytes. a-c, C1*s* (a), O1*s* (b) and F1*s* (c) spectra of NCM811 cathodes harvested from 1 M $LiPF_6$-EC/DEC electrolytes at etching time of 0 min and 12 min. **d-f**, C1*s* (d), O1*s* (e) and F1*s* (f) spectra of NCM811 cathodes harvested from ct/cc-0.82 electrolytes at etching time of 0 min and 12 min. **g-h**, Ex situ FT-IR analysis of NCM811 cathodes at different charge and discharge states 1 M $LiPF_6$-EC/DEC electrolytes (g) and ct/cc-0.82 electrolytes (h). For the NCM811 cathode harvested from 1 M $LiPF_6$-EC/DEC electrolytes, it was obtained after operating for 68 cycles at current density of 0.5C (100 mA g^{-1}), and the cathode is in a fully discharged state. For the NCM811 cathode harvested from ct/cc-0.82 electrolytes, it was obtained after operating for 100 cycles at current density of 0.5C (100 mA g^{-1}), and the cathode is in a fully discharged state. The charging cut-off voltage for both electrolytes is 4.8 V.

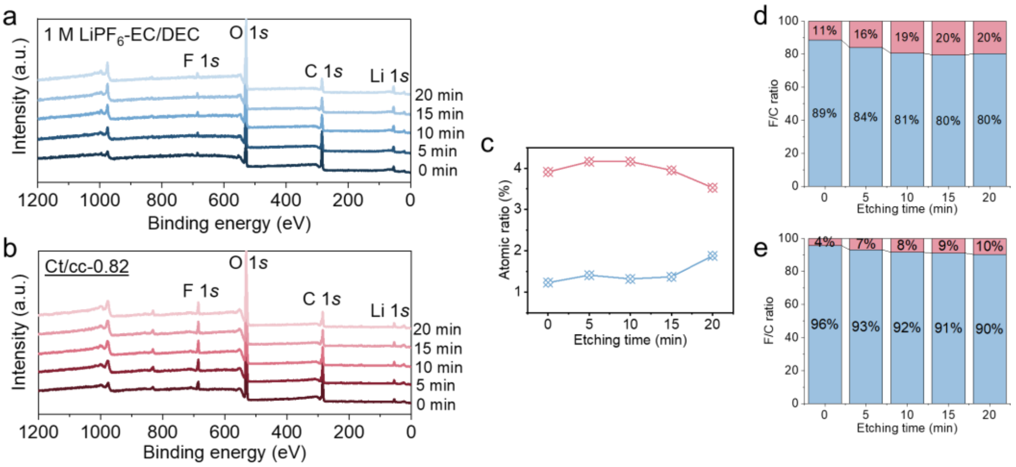

Supplementary Figure 24 | XPS analysis of NCM811 cycled in different electrolytes. a-b, XPS spectra of cycled NCM811 in 1 M $LiPF_6$-EC/DEC electrolytes and ct/cc-0.82 electrolytes with different etching times. **c**, XPS depth profiles of F in 1 M $LiPF_6$-EC/DEC electrolytes and ct/cc-0.82 electrolytes. **d-e**, F/C atomic ratio changes with etching time in these two electrolytes. For the NCM811 cathode harvested from 1 M $LiPF_6$-EC/DEC electrolytes, it was obtained after operating for 68 cycles at current density of 0.5C (100 mA g^{-1}), and the cathode is in a fully discharged state. For the NCM811 cathode harvested from ct/cc-0.82 electrolytes, it was obtained after operating for 100 cycles at current density of 0.5C (100 mA g^{-1}), and the cathode is in a fully discharged state. The charging cut-off voltage for both electrolytes is 4.8 V.

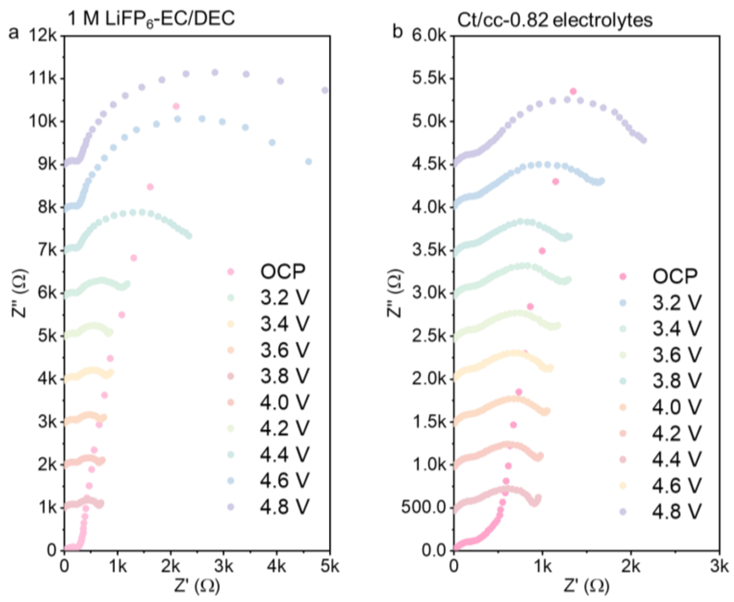

Supplementary Figure 25 | In-situ EIS during charging. a, Using 1 M $LiPF_6$-EC/DEC electrolytes. **b**, Using ct/cc-0.82 electrolytes.

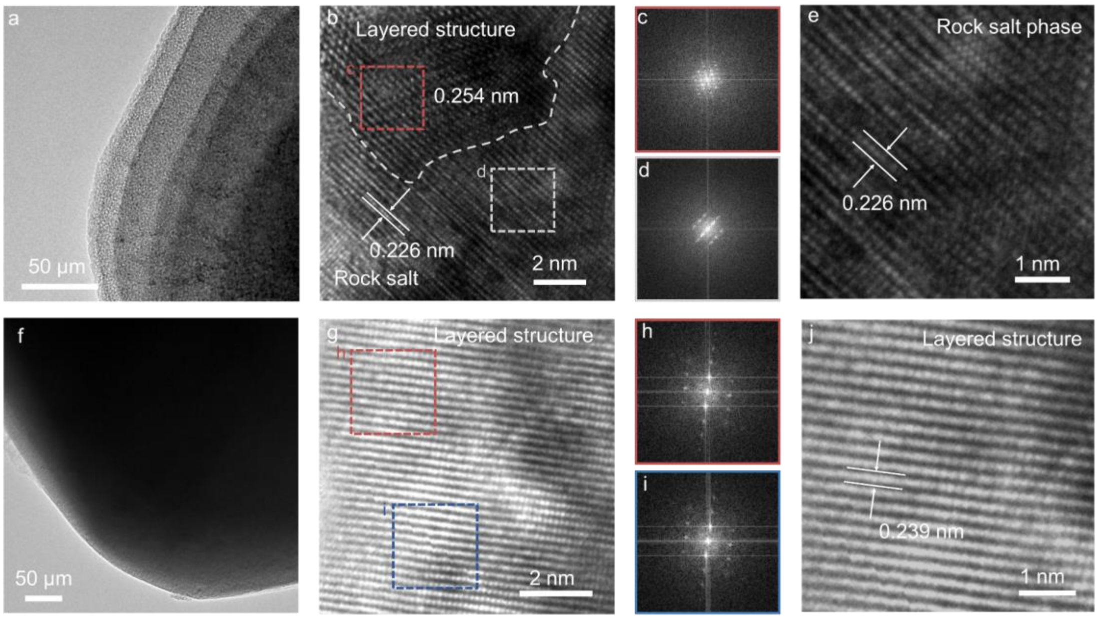

Supplementary Figure | 26 Structural evolution of NCM811 operating under high voltage. a-b, The TEM images of NCM811 cathodes harvested from 1 M $LiPF_6$-EC/DEC. **c-d**, FFT images of the region outlined in (b). **e**, Magnified TEM image. **f-g**, The TEM images of NCM811 cathodes harvested from ct/cc-0.82 electrolytes. **h-i**, FFT images of the region outlined in (g). **j**, Magnified TEM image. For the NCM811 cathode harvested from 1 M $LiPF_6$-EC/DEC electrolytes, it was obtained after operating for 68 cycles at current density of 0.5C (100 mA g^{-1}), and the cathode is in a fully discharged state. For the NCM811 cathode harvested from ct/cc-0.82 electrolytes, it was obtained after operating for 100 cycles at current density of 0.5C (100 mA g^{-1}), and the cathode is in a fully discharged state. The charging cut-off voltage for both electrolytes is 4.8 V.

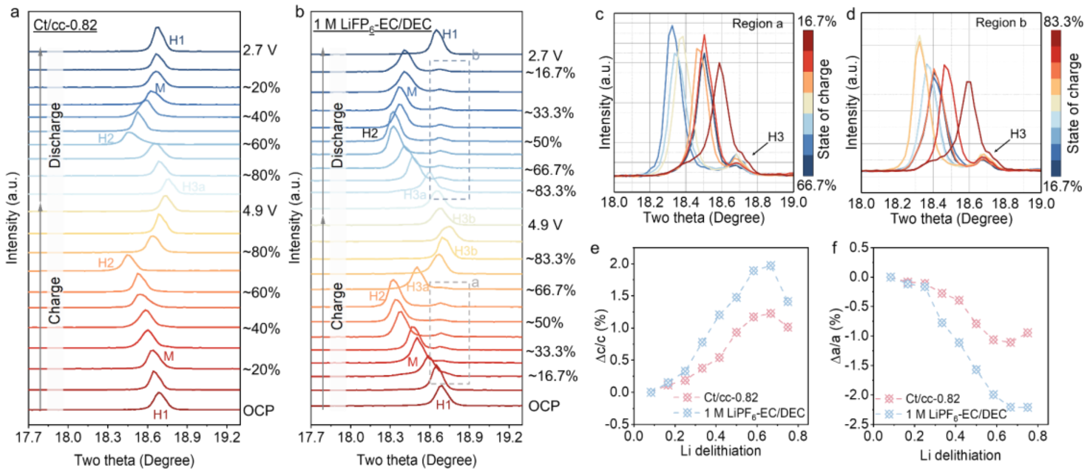

Supplementary Figure 27 | Ex situ XRD tests of cathode using different electrolytes. a-b, Ex situ XRD evolution of NCM811 in ct/cc-0.82 electrolytes (a) and 1 M $LiPF_6$-EC/DEC electrolytes (b) at 11th cycle. **c-d**, The enlarged region of a and b outlined in (b). **e-f**, Lattice parameters of *a* and *c* axes change during the 11th charging in 1 M $LiPF_6$-EC/DEC (e) and ct/cc-0.82 electrolytes (f). The corresponding state of charge (SOC) is marked on the right side of Figure a and Figure b.

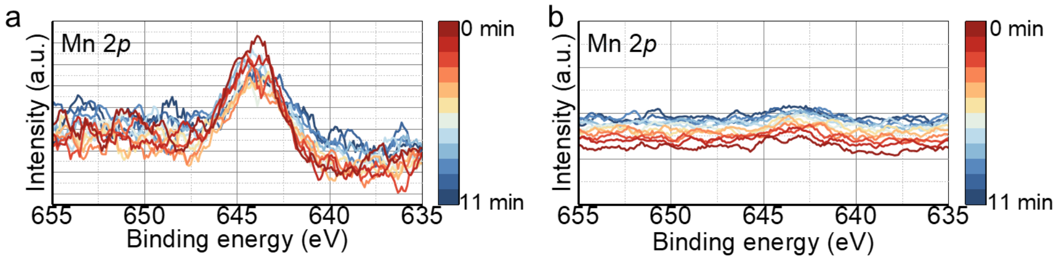

Supplementary Figure 28 | Mn 2*p* signal profiles of SEIs formed in different electrolytes. **a**, Using 1 M $LiPF_6$-EC/DEC electrolytes. **b**, Using ct/cc-0.82 electrolytes.

These values in radar chart (Fig. 1c) were calculated based on 17 M LiFSI-DMC (high cis-trans) electrolytes, 1 M $LiPF_6$-EC/DEC (ester electrolytes) and 1 M LiFSI-DME (ether electrolytes). To generally rate these electrolytes, we tested the relevant performance of the three electrolytes. For the safety, potential window, stability against cathode, stability against Li anode and anti-corrosion performance, high cis-trans electrolytes have the best performance, which is defined as the maximum. For the ionic conductivity, the ether electrolytes have the best performance, which is defined as the maximum. The radar chart indicates the top performance for each parameter in the field, not the combined performance of any specific electrolytes.

Supplementary Table 1. High voltage NCM811 performance comparison

Positive electrode	Mass fraction of positive (wt%)	Electrolytes	Mass loading (mg cm^{-2})	Electrolyte volume (μL)	Temp (°C)	Negative electrode	Cut-off voltage (V)	Applied current (mAg^{-1})	Lifespan	Capacity fading rate	References
NCM811 (Single crystal)	80	ct/cc-0.82	1-2	50	25	Li metal	4.8	100	1000	0.031%	This work
NCM811 (Polycrystal)	94	1 M LiFSI/DMTMSA	7.5	20	25	Li metal	4.7	100	100	0.119%	*Nat. Energy* 2021, 6, 495–505.
NCM811	80	1 M $LiPF_6$-FEC/BTC	8~8.5	30	-	Li metal	4.8	100	120	0.156%	*Energy Environ. Sci.*, 2022,15, 2435-2444.
NCM811 (Single crystal)	80	1 M $LiPF_6$ MTFP/EMC/FEC	-	-	25	Li metal	4.8	40	100	0.188%	*Front. Energy Res.* 2022, 10, 973336.
Co and Ti co-modified NCM811	70	-	1-2	-	25	Li metal	4.7	-	400	0.065%	*ACS Appl. Mater. Interfaces.* 2021, 13, 17707–17716.
NCM622	80	1 M $LiPF_6$-EC/EMC with 5% PFPBA	4.2	30	Room temperature	Li metal	4.8	1000	150	0.162	*Angew. Chem. Int. Ed.* 2023, e202300057.
NCM811 (Single crystal)	80	1.2 M $LiPF_6$-TMS/FEC	-	80	Room temperature	Li metal	4.7	100	200	0.1%	*Chem. Eng. J.* 2023, 465, 142907.
NCM811 (Polycrystal)	80	1 M LiTFSI-HFE/FEC	3-4	-	25	Li metal	4.7	100	150	0.1%	*J. Power Sources* 2022, 517, 230683.
